# ‘You know what, I’m in the trend as well’: understanding the interplay between digital and real-life social influences on the food and activity choices of young adults

**DOI:** 10.1017/S1368980022000398

**Published:** 2022-08

**Authors:** Jodie Leu, Zoey Tay, Rob M van Dam, Falk Müller-Riemenschneider, Michael EJ Lean, Charoula Konstantia Nikolaou, Salome A Rebello

**Affiliations:** 1 National Centre for Epidemiology and Population Health, Research School of Population Health, The Australian National University, Canberra, ACT, Australia; 2 Saw Swee Hock School of Public Health, National University of Singapore, Tahir Foundation Building, 12 Science Drive 2, #10–01, 117549 Singapore, Singapore; 3 Department of Exercise and Nutrition Sciences, Milken Institute School of Public Health, The George Washington University, Washington, DC, USA; 4 Yong Loo Lin School of Medicine, National University of Singapore, Singapore, Singapore; 5 School of Medicine, Dentistry & Nursing, University of Glasgow, Glasgow, Scotland; 6 Edgar Diabetes and Obesity Research, Dunedin School of Medicine, University of Otago, Dunedin, New Zealand; 7 Natural Resources Institute, Faculty of Engineering and Science, University of Greenwich, Kent, UK; 8 Division of Biostatistics and Bioinformatics, Graduate School of Public Health, St. Luke’s International University, Tokyo, Japan

**Keywords:** Young adults, Diets, Physical activity, Social environments, Social media, Choices

## Abstract

**Objective:**

To understand young adults’ perceptions of online and real-life social influences on their food and activity choices.

**Design:**

A qualitative study involving 7 focus groups. Thematic analysis using both deductive and inductive techniques were performed.

**Setting:**

A polytechnic and a university in Singapore.

**Participants:**

A total of 46 full-time students, 19–24 years of age.

**Results:**

Participants revealed that social media meets multiple needs, contributing to its ubiquitous use and facilitating content spread between social networks. Food-related content shared on social media were mostly commercial posts, marketing foods and eateries showcasing price promotions, emphasising sensory properties of foods or creating narratives that activated trends. Subsequently, real-life social activities frequently revolve around marketed foods that were not necessarily healthy. In contrast, physical activity posts were rarely being followed up in real life. Portrayals describing a toxic gym culture could contribute to negative perceptions of peers’ physical activity posts and a disinclination towards sharing such posts. Participants expressed that close, supportive social networks in real life strongly influenced initiating and maintaining healthy lifestyles. However, in a society that highly values academic achievements, participants prioritised studying and socialising over healthy eating and physical activity.

**Conclusions:**

Overall, our findings reveal that virtual and real-life social influences have complex interactions affecting Asian young adults’ behavioural choices and should be considered when designing interventions for this group. Regulations related to the digital marketing of unhealthy food, and improving the availability, accessibility and affordability of healthier food options, particularly in the foodservice sector, would be of value to consider.

The importance of healthful eating and physical activity for preventing obesity and decreasing disease risk is well established^(1)^. However, young people in Singapore, as elsewhere, tend to adopt dietary behaviours, such as not meeting recommended daily servings of fruit and vegetables^(2)^ and frequently eating at ‘Western’ fast-food venues^(3)^, associated with excessive weight gain^(4,5)^. While 13·1 % of Singaporean adults in their 20s (18–29 years) have high-risk BMI of ≥27·5, according to BMI cut-offs for Asian populations^(6)^, this proportion almost doubles to 22·4 % for adults who are in their 30s (30–39 years)^(7)^. Similar patterns of weight gain have been observed internationally such as in Europe, Australia and the USA^(8)^. As losing weight and maintaining weight loss is challenging, preventing excessive weight gain is important for having a healthy body weight. Emerging adulthood, a developmental period typically defined as an age between 18 and 25 years, is also a time of increasing autonomy, when social identities are explored, and habits which are likely to be long standing in nature develop^(4,5)^. High value is also placed on fitting in and finding friends as young people transition from secondary to tertiary education^(9,10)^. This period, therefore, presents an important opportunity for health-directed dietary and physical activity interventions.

The role of environmental factors, including the affordability, availability, accessibility and marketing of food, that can influence dietary behaviours are increasingly recognised^(11,12)^. Growing evidence also suggests that social influences may have a strong impact on young adults. Social networks can influence food and activity choices via interacting pathways of peer influence^(13–15)^, social support^(13,14,16)^ and social norms^(17)^. Peers can positively affect dietary practices and physical activity by supporting health-promoting practices to achieve weight loss goals^(15)^, and by extending invitations to participate in sports or cooking meals together^(13,14)^. Conversely, peers may discourage health-promoting practices by engaging in sedentary social activities or dissuading physically active social pursuits^(13,14)^. Social support, including the types of information and resources shared within social networks, can also influence the dietary and activity behaviours of network members^(13,14,18)^. Further, social norms influence multiple aspects of consumption such as whether it is socially acceptable to dine alone and to use mobile devices during mealtimes^(17)^. Social norms also influence dietary choices as people tend to model their food choice and consumption based on their companions, such as mirroring others’ portion sizes and food choices and women eating less in the presence of men^(19)^.

These interacting pathways of peer influence, social support and social norms which play out in real life may be amplified in digital spaces as young adults are concerned with how they are perceived on social media and are receptive to social influences. As social media engagement becomes more pervasive, social networks have expanded and can have transformative effects. Globally, social media use is highly prevalent, with those aged 18–34 making up the majority of social media users across various social media platforms^(20)^. Singapore is a multi-ethnic, high-income, urban nation located in Southeast Asia with a representation of 3 major Asian ethnic groups, Chinese, Indians and Malays. Eating out is a pervasive norm with 77·3 % of Singaporeans usually having either breakfast, lunch or dinner outside and is supported by a highly accessible food environment catering to a diverse range of palates and budgets^(3)^. In Singapore, 84·4 % of the population were active social media users in 2021^(20)^. These aspects suggest that social networks, both real and virtual, can have a strong influence on the food and activity choices of the Singaporean population. Most studies that explore this phenomenon are based on Western populations^(17,19,21)^ with some in university settings^(13,14)^ and few in Asian settings.

This study is part of a larger qualitative cross-cultural study on social, ethical and moral perspectives of young adults around the consumption of food and physical activity in Europe and Singapore. We have investigated young adults’ perceptions of the social environment on their food and activity choices through a qualitative approach, in an English-speaking Asian context. The findings may help inform current and future health interventions directed towards young adults.

## Methods

### Study design

A qualitative study design was utilised to explore and gain in-depth insights of factors that influence young adults’ dietary and physical activity practices^(22)^. Purposive sampling was used to facilitate the recruitment of participants similar in age and life experiences who were knowledgeable and experienced with the phenomena being studied^(22)^. As the study is exploratory in nature, the use of focus group discussions (FGD) allowed for the collection of a wide range of viewpoints and subsequent exploration of expressed viewpoints through synergistic interactions between group participants^(22)^. The 32-item consolidated criteria for reporting qualitative studies (COREQ) checklist was followed for the preparation of this paper^(23)^.

### Study setting

The study was conducted in English amongst students from the National University of Singapore (NUS) and Republic Polytechnic (RP). Differing from polytechnics in the USA and the UK, most Singaporean polytechnics admit students who have completed lower secondary schooling at around 16 years of age. RP is a diploma-granting higher education institute with an enrolment of 13 566 students^(24)^, most of whom live off-campus, typically at parental homes. NUS is a degree-granting institute with approximately 40 000 students, a quarter of whom are postgraduates^(25)^. Approximately 10 000 students live on campus, usually during their first and second years of undergraduate study.

### Participant recruitment

To be included, participants had to be full-time students at either RP or NUS, at least 18 years of age, own and use a smartphone and be Singaporean citizens or permanent residents. We excluded participants who were pregnant or breastfeeding.

We purposively recruited students at NUS using email blasts and at RP using onsite recruitment and friend referrals. A total of 163 students (62 men and 101 women) expressed their interest via email or text messages and 112 students (47 men and 65 women) were screened while 51 students (15 men and 36 women) were not screened due to the following reasons: decided not to participate following their expression of interest, unable to be reached following their expression of interest or FGD numbers had been reached. Of the screened participants, 103 students (44 men and 59 women) were eligible, and 9 students (3 men and 6 women) were not eligible. We were unable to schedule discussions for 57 students (25 men and 32 women) who were eligible. This was due to: them not wanting to join the study following their expression of interest, scheduling conflicts or a decision we made that the study had reached thematic saturation. A total of 46 students (19 men and 27 women) of mixed academic disciplines participated in the study. Recruitment ended when discussions between JL, ZT and SAR deemed data saturation had been reached.

### Study procedure

Prior to the start of FGD, participants were asked to fill out a short demographic survey to collect information on their age, height, weight, gender, ethnicity, marital status, institution of study, faculty, course of study, year of study and their use of social media platforms (Facebook, Instagram, Twitter and Weblogs).

FGD were conducted between March and May 2017 at designated conference rooms at the institutions. Separate discussions were conducted with men and women as some topics such as body image were considered sensitive. Among men (*n* 19), 2 FGD with 8 and 7 participants took place at NUS and 1 FGD with 4 participants took place at RP. Among women (*n* 27), 2 FGD took place at NUS with 7 and 8 participants and 2 FGD took place at RP with 6 participants each. Where possible, friends were not scheduled in the same focus group. The discussions were facilitated by JL and ZT, who were female postgraduate students trained in qualitative methodologies and had no prior relationships with the participants. All participants provided verbal informed consent prior to participation.

A discussion guide was developed and piloted by the study team, and included topics on social media use, factors influencing food and physical activity behaviours and mobile application preferences. For the purposes of this paper, social media is defined as internet-based platforms where users can create individual profiles, contribute and access searchable digital content (e.g. content intended to inform, entertain or sell products) and form online connections with other social media users^(26)^. Discussions lasted 90 min on average. Debrief sessions took place after the focus groups between JL, ZT and SAR. Data saturation was discussed between JL, ZT and SAR and seemed to have been reached despite a small number of focus groups. All FGD were audio-recorded, transcribed verbatim by a contracted transcription company and verified by focus group facilitators. Member checking was not conducted to limit the burden on participants^(27)^.

### Thematic analysis

**Fig. 1:**
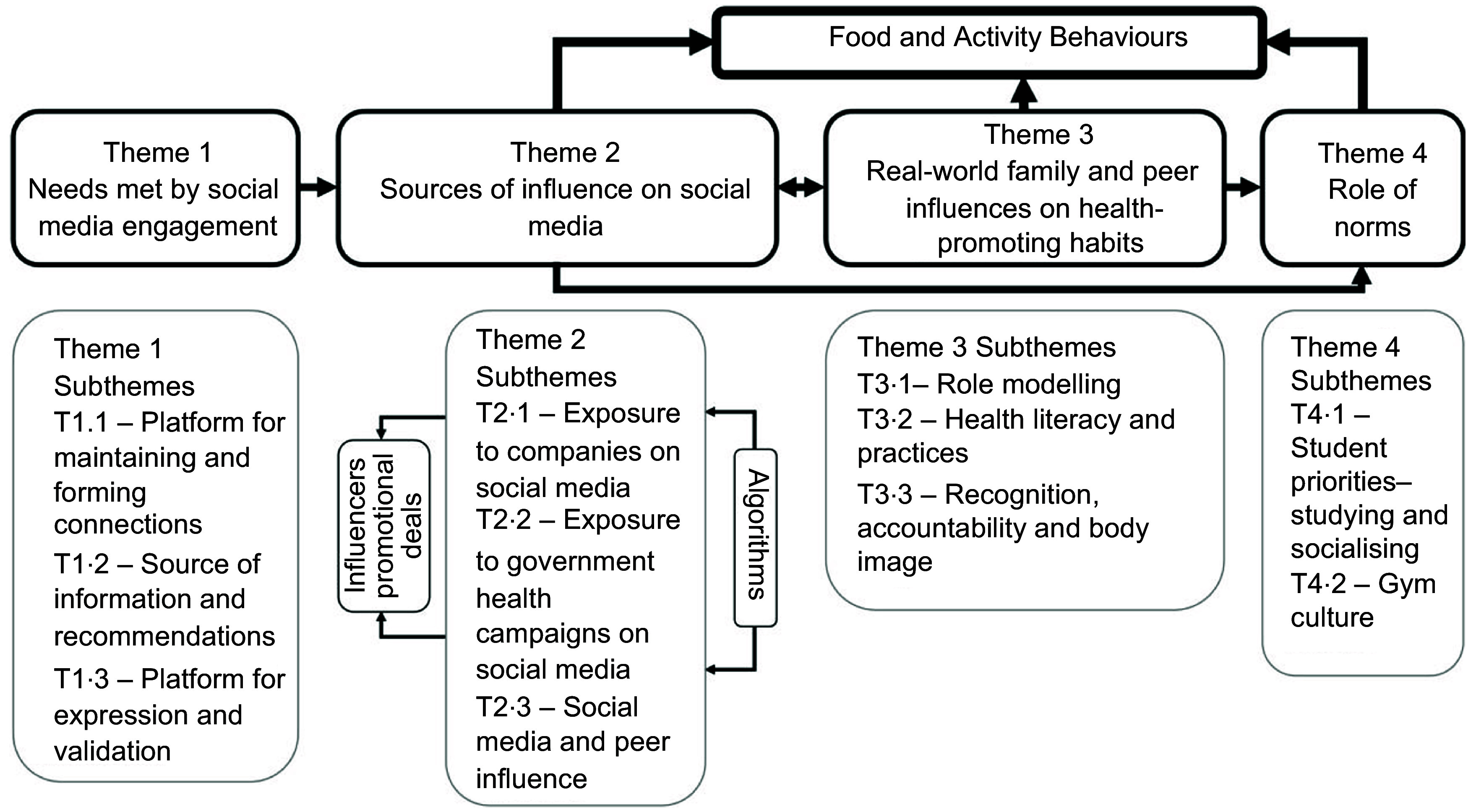
Thematic relationship to illustrate the role of real and virtual social influences on food and activity behaviours

Each transcript was reviewed and coded using thematic analysis which allows for using both inductive and deductive approaches^(28)^. With the deductive approach, transcripts were coded for themes that were identified from the literature^(13,14,19,21)^. With the inductive approach, additional themes were added if relevant during the coding process allowing for the inclusion of unanticipated themes. Each transcript was coded by JL and ZT independently. Upon the completion of coding, JL and ZT compared, reviewed and finalised themes. Further discussions of the themes and the relationships between them took place between JL, ZT and SAR to develop a mind-map that illustrated the relationships between themes (Fig. 1)^(28)^. Nvivo software (version 11, QSR International, Australia) was used to organise and analyse the transcripts. To maintain confidentially, all participants have been assigned codes.

**Fig. 2:**
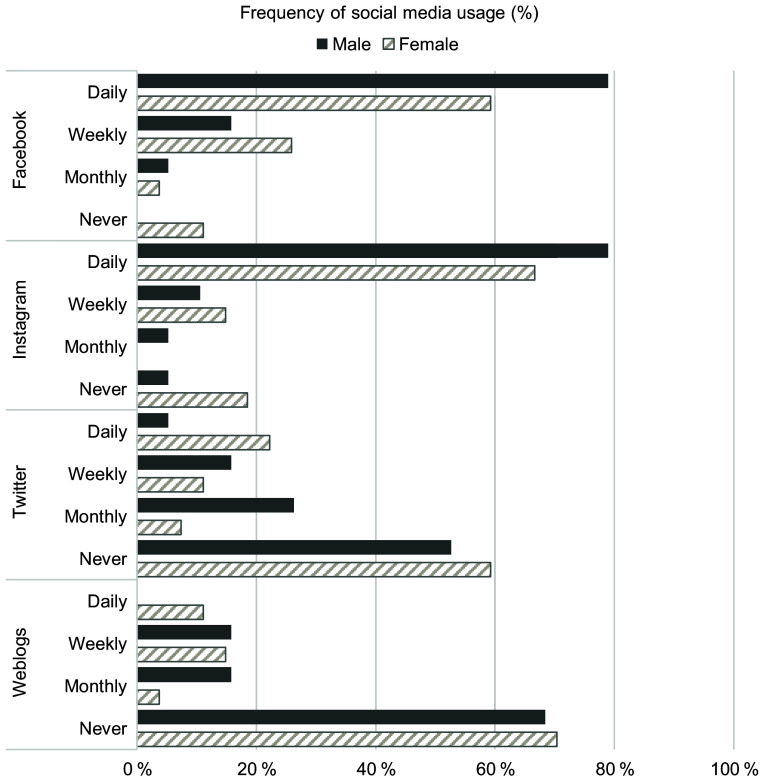
Frequency of social media use of young men (*n* 19) and women (*n* 27)

## Results

### Participant characteristics

A large proportion of participants were ethnic Chinese (80·4 %, *n* 37) which is broadly reflective of the national profile (Chinese: 74·3 %, Malays: 13·5 %, Indians: 9·0 %, Others: 3·2 %)^(29)^. All participants were studying full time, of ages ranging 19–24 years old, and all were unmarried (Table 1). Participants represented a wide spread of study majors and years of tertiary education. Close to 40 % (*n* 18) of participants were overweight according to Asian criteria (BMI ≥ 23 kg/m^2^) (Table 1). All participants engaged with at least one social media platform with Facebook being the most popular (*n* 43, 93·5 %) followed by Instagram (*n* 40, 87·0 %), Twitter (*n* 20, 43·5 %) and Weblogs (*n* 14, 30·4 %). Facebook and Instagram had high usage (Fig. 2). Participants were all able to speak and express themselves freely in English with the occasional use of colloquial Singaporean English (Singlish).

**Table 1:** Participant characteristics across 7 focus groups by gender

	Total (*n* 46)		Male (*n* 19)		Female (*n* 27)	
Number of focus groups	7		3		4	
	Mean	sd	Mean	sd	Mean	sd
Age (years)	20·9	1·8	21·6	1·9	20·4	1·6
BMI (kg/m^2^)	22·1	3·0	23·2	3·0	21·3	2·8
	*n*	Percentage	*n*	Percentage	*n*	Percentage
< 18·5	1	2·2	0	0·0	1	3·7
18·5–22·9	27	58·7	8	42·1	19	70·4
23–27·5	15	32·6	9	47·4	6	22·2
≥ 27·5	3	6·5	2	10·5	1	3·7
Ethnicity	*n*	Percentage	*n*	Percentage	*n*	Percentage
Chinese	37	80·4	14	73·7	23	85·2
Malay	2	4·3	1	5·3	1	3·7
Indian	4	8·7	3	15·8	1	3·7
Other	3	6·5	1	5·3	2	7·4
Marital status	*n*	Percentage	*n*	Percentage	*n*	Percentage
Single	46	100·0	19	100·0	27	100·0
Year of study*	*n*	Percentage	*n*	Percentage	*n*	Percentage
1 (RP and NUS)	11	23·9	5	26·3	6	22·2
2 (RP and NUS)	15	32·6	6	31·6	9	33·3
3 (RP and NUS)	11	23·9	4	21·1	7	25·9
4 (NUS only)	8	17·4	4	21·1	4	14·8
Course of study	*n*	Percentage	*n*	Percentage	*n*	Percentage
Business†	4	8·7	3	15·8	1	3·7
Design and environment	1	2·2	0	0·0	1	3·7
Law	2	4·3	1	5·3	1	3·7
Arts and social science‡	12	26·1	5	26·3	7	25·9
Applied science§	20	43·5	8	42·1	12	44·4
Life science	5	10·9	2	10·5	3	11·1
Physical science‖	2	4·3	0	0·0	2	7·4

* Numbers do not add to 100 % due to missing data. Polytechnics usually offered 3-year diplomas while NUS usually offered 4-year degrees.

† Includes: Accountancy, Business Administration.

‡ Includes: Communications and New Media, Economics, Geography, Political Science, Psychology, Sociology, Undeclared Arts and Social Science student.

§ Includes: Medicine, Pharmacy, Health Management and Promotion, Outdoor and Adventure Learning, Biomedical Science, Computer Science (includes Information Technology related courses), Engineering.

‖ Includes: Environmental Science.

Percentages may not add up to 100 % due to rounding.

Our findings are presented under 4 main themes as shown in Fig. 1: needs met by social media engagement, sources of influence on social media, real-world family and peer influences on health-promoting habits and the role of norms. The relationships between each of the themes and how they ultimately influence young adults’ food and activity behaviours are described under each of the themes.

### Theme 1. Needs met by social media engagement

Most participants had social media accounts that they used daily, with Facebook (daily use: men: 78·9 % ; women: 59·2 %) and Instagram (daily use: men: 78·9 % ; women: 66·7 %) being the most popular (Fig. 2). This high use was motivated by a need to connect and be informed of events and interests in the lives of family and friends and of news in the world. Through social media, participants interacted with people within and outside their close social networks to share content and promotional deals, and to pick up new skills. Illustrative quotes for this theme are presented in Table 2.

**Table 2:** Quotes for theme 1: needs met by social media engagement

Sub-Theme	Illustrative quotes
T1·1 Platform for maintaining and forming connections	So, the way I use social media is not indeed to actually like put myself out there or like um, update people about myself. But more of like viewing what other people has been doing. Yeah, and more of like taking in information. – F04, female, 19, Infocomm Security Management
	Um, okay, for me I usually update Instagram when there are, like, occasions, or, like, events that happen in my life, and I just want to share it, because I feel like, um, you think, like, social media helps you, it’s not just like for other people to update you but also, like, you can easily update, like, a lot of people around you without, like, personally messaging every single one of them, or, like, hey, this thing happened in my life, you know, it’s like – when you just post it, like, it’s easier to facilitate this kind of information. So, for me that’s why I use Instagram for and, sometimes also just ’cause I find some photos nice, then I just want to post it, just to share it with my friends. And then Facebook is usually – I just – I don’t really post anything, but I just, like, share, what I find, like, interesting or funny that, like, other friends have shared before. Yeah – C04, female, 19, Psychology
	I follow the Instagram accounts of my, of the Olympic medalists for my sport. So, just to check them out. So, uh, to see them skills-wise and try to imitate them. – B08, male, 21, Life Science
T1·2 Source of information and recommendations	I actually like bookmarked a lot of them. [other participants laughed] Like “get toned in like 30 days” that kind. I have so many of them on my Facebook like bookmark list, but I never go back to them. [background laughter and other participants vocalizing agreement] – A05, female, 21, Life Science
	Um, for me, I rarely notice um apps related to fitness, but maybe food, but usually I don’t really respond to these apps unless, um, there’s promotion, or I’m interested in it, or my friends have talked about it, yeah. – C07, female, 23, Economics
	I’m not sure if y’all know [popular local lifestyle blogger]. Like when the *Chizza* [Chizza is a portmanteau of ‘chicken’ and ‘pizza’. This product was sold at Kentucky Fried Chicken in Singapore] just came out and she was eating it, I wanted to try it. Yeah. So that was one of the factors of advertisements that was quite good I think… Lucky I didn’t [try] ’cause my friend told me it’s not nice. – A02, female, 23, Project and Facilities Management
T1·3 Platform for expression and validation	I think, sometimes when I see those café stuff, right, I feel like people don’t always go there just to try them, but out of social pressure. It’s like everybody’s going there, so it’s like I have to go there and try also, otherwise I would be left out. Then people will be talking about it like Art Box [A huge flea market with more than 300 fashion and food stalls that happened from April 14th – 23rd, 2017]. Like, initially I don’t think a lot of people was interested in it. Most people go out of social pressure because all their friends are posting on Instagram, like “I am going to Art Box, trying new foods”, so like, okay, you know what, I should go and try also. Even though people say it sucks, but everybody is going, so I just go… Yeah, it’s like to show people that “you know what, I’m in the trend as well”. – E05, female, 19, Environmental Science
	I have a friend, who’s a bodybuilder, and he always posts like his own selfies on Facebook and he puts some stupid caption that I don’t even know what, how does that relate to the photo? – D07, male, 24, Political Science
	Yeah, and they also have, like, uh trainer, they will like promote themselves on Instagram, like they will suddenly pop up at the explorer thingy. Yeah, so like, I follow them ah, like… It motivates me to like, exercise and eat healthy. And like, when you see your friends also, like they, their body, or like nice right? Yeah, then like, make me feel down also lah, sometimes. But it also boost you up ah actually. Why you laugh? [participants laughed] Yeah, it actually motivate you ah, to like, have that body you know. Like yeah. – F04, female, 19, Infocomm Security Management
	It gets a bit annoying. I don’t know, I see Facebook and oh your friend just completed a 6km run and I’m just like…hmmm…[Participants laughed in the background hence the complete sentence cannot be heard clearly] then like. – B01, male, 23, Life Science I don’t see the need to publicize my, whatever you have done ah. – B02, male, 22, Economics Same. Like it gets annoying when you see people posting. Like, okay, so what? What are you trying to prove? But yeah, I think it depends on the individual lah. Like are you doing it for yourself or are you doing to show people that you are doing it? – B08, male, 21, Life Science I…I think that… Actually, let’s take Nike running or any of those other running apps for examples, you can add friends within the app. So, if there are people who are actually interested in that information, they would probably add you as a friend. You guys can talk about it and add through the app, as opposed to on Facebook. Because the people on Facebook don’t care if I ran 9 km last night. [some laughs from other participants] – B04, male, 21, Business Administration
	Um, I think, for myself, like I see a lot of the, like those home boxing clubs, or like gym kind of, free trial classes that they always offer at the beginning, and things like that, and then, uh, for me um, I generally don’t respond unless, like, I really have an inclination towards it lah, like, [unclear 00:14:39], yeah, or unless it’s something really novel and, like, hey like I could try this out, then I would like just respond… Uh, there’s this thing, there’s this um, new workout place called B-Bounce [Singapore’s first dedicated rebounding fitness studio], so basically what they did, what they do is, like, you work out on the trampoline, and then it’s something, like, really interesting, I mean, it’s not like trampoline park where you just bounce around, but, like, you follow, like, what the instructor does, and it actually like is really quite effective, I feel. [laughs] Yeah…. Yeah, I tried it. Like, I tried with my friend, so basically this friend, I will always like to share these kinds of things between ourselves. – C03, female, 21, Business Administration

#### Subtheme T1·1 Platform for maintaining and forming connections

Keeping in touch with friends and family on social media by being up to date with events and interests was one of the main reasons why the participants had social media accounts even if they were not visibly active on their own accounts. Through their digital persona, participants sustained relationships and created social capital through interactions with their social networks. Participants also used social media to connect with people and entities outside their social networks especially when seeking new information thus effectively expanding their social networks. For instance, participants recalled following a personal trainer on social media to view free exercise programmes that they may also share with their social networks.

#### Subtheme T1·2 Source of information and recommendations

Participant responses suggest that the content they are exposed to often serve to pique their interest in certain ideas based on which they may conduct their own research. While participants recognised the limitations of digital sources of information, they also described ways of managing this, as D01 (male, 21, chemical engineering) describes, *‘Usually I’ll source a few different websites, ’cause a lot of uh, sensationalist news now.’* Although participants were often suspicious of the veracity of the content on social media, they admitted that there were also times when they shared information based solely on headlines. Nonetheless, several participants reported using social media sites as inspiration for eating healthfully, cooking or being active, even though they may not always follow this through with long-term behaviour change.

Social media served as a platform where participants actively searched and shared recommendations on activities and price promotions for future social occasions. Some examples voiced by participants included ‘buy one get one free’ deals from a coffee shop chain, gym promotions and discounted buffets. The types of information shared also generally reflected the interests of their friends in a social group. Young adults in Singapore also turn to social media accounts of influential and prolific bloggers for information on food and activities. While recognising that some of these may be industry-sponsored posts, they were nevertheless regarded as being somewhat persuasive. Opinions of friends, however, were more trusted when these differed from influencers. Consequently, content shared with friends had the effect of being a personal recommendation, which may have a stronger influence than marketing advertisements as participants tend to trust posts from their social network.

#### Subtheme T1·3 Platform for expression and validation

Social media serves as a platform for young adults to express themselves and showcase their experiences. Some participants talked about how sharing information on social media was the primary motivator for going to food places or events, many of which were planned as part of social gatherings. A degree of ‘fear of missing out’ also provided motivation for participants to eat specific foods and participate in activities that were trending with their friends. By sharing their own experiences of trendy activities on social media, participants sought to gain validation from their peers. In general, there seems to be a need to stay informed, and a sense of unease related to missing out on news, events in friends’ lives, trends, promotions and recommendations. However, friends’ posts were not always positively regarded, and achievements such as gym workouts or completing a run were viewed by some participants as being pretentious or ‘*annoying*’. Seeking validation from others or facilitating accountability were perceived as motivations for why peers share physical activity achievements. Participants also seemed disinclined to post their own physical activity achievements widely, describing this information as something they might not want anybody to know, unless it was with a specific group, such as a training group that shared the same interest. Sometimes, posts by peers or fitness instructors may motivate participants to exercise, though at times, some participants described feeling negatively due to comparing themselves with the person in the post.

### Theme 2 – Sources of influence on social media

Whilst on social media platforms, participants described encountering various sources of influences including from institutional entities such as food companies, gyms and government organisations and influences from peers. Illustrative quotes for this theme are presented in Table 3.

**Table 3:** Quote for theme 2: sources of influence on social media

Sub-Theme	Illustrative quotes
T2·1 Exposure to companies on social media	Especially McDonald’s, right, even if you don’t have it for a very long time. And then it release a new burger and your friend was like, “have you tried their new burger?” [participants laughed]. Well looks like I’m going there. Or they will *jio* [Singaporean slang for “invite”] you go. And, and it works, you know. It works so well. Uh, every time they do this promotional campaign, a bunch of people go just for the hype. [A few participants making “mm” sounds in agreement to what was said] … They [the marketing companies], they know it. They’re cashing in on it. There weren’t so many specialty burgers in the past. Now there’s like a few every year. – D01, male, 21, Chemical Engineering
	So, because the companies, since they are big companies, and all, they will actually pay people to try their stuff or their… ‘Cause, um, I do have some friends who are like influencers, but, although not mainly, not mainly food. They are like, they are people who actually take very beautiful photos and things like that. The companies would actually contact them, like, “hey, we have a party for you at this time, this place. So, uh, come and then, you know, we can reveal about us”, things like that. Then, you know, most often than not, they’ll write very pleasing reviews and things like that. – G04, male, 18, Information Technology
	Yeah, food bloggers, and they will influence how we eat overall, yeah. Because most people nowadays, we consume a lot of social media, so if person A says, so if person A says, like, say the main person who blogs right, say, hey this was very good, we should all try it. Then the next person who hops on to, like, the blog site, then they would go try it, and then this chain just continues on to more and more people. – G02, male, 19, Information Technology
	Normally how they shape the marketing campaign is based on the public sentiments. So, if you believe that their marketing strategies are not very ethical right, it’s because there are vulnerabilities in how the society thinks and they’re just tapping on it. So, if, if you don’t, we want to weed out the bad or unethical marketing strategies, right, the people have to change their mind-set first. – D04, male, 24, Mechanical Engineering Mm. So what’s your personal take towards this? Like, how do you respond, then, to such marketing strategies? – Focus Group Facilitator I mean, it’s, it’s… If you don’t want them to do this, you have to… become more witty and to know what’s right and what’s wrong lah. Because if the marketing campaign doesn’t stick, right, it will show in the sales, in all the figures that they have lah. Usually, they will have a… after they have an advertising campaign there will always be numbers, then they will do surveys on how the reception is like. So, it’s actually the people lah, if you ask me. Like, to put another perspective lah, it’s not necessarily their fault. The people are the ones who have the wrong, uh, perception of certain things. And they’re just making use of that weakness ah, that people have, yeah. – D04, male, 24, Mechanical Engineering So how do you personally feel towards that? – Focus Group Facilitator Think uh, the smart people will know lah. That’s what I feel lah. It’s only the like more gullible ones who will fall for these kind of things. – D04, male, 24, Mechanical Engineering
	I don’t really look at these kinds of advertisements unless, unless it’s for promotions. Like, food promotions. Like, there was a period of time when there was, like, a lot, like, Uber Eats [an online meal ordering and delivery platform] discount codes, then they [the company] posted, then everyone just went, like you know, take advantage of the situation. – C04, female, 19, Psychology
	But then, um, I guess sometimes when you like scroll through Insta [Instagram] or what, then they have those adverts, you know those mini-adverts that they started doing. Then sometimes got food or like certain commercials on going out all that kind, yeah then I will, I would click and see ah. Yeah… sometimes…Like there was the… The Manhattan Fish Market was showing, and KFC also had the, you know those online coupon thingies…Yeah yeah, so they had that on Insta, so I was like, okay, I’ll get mine now. – D05, male, 21, Economics
	Uh my gym membership from Anytime Fitness…Yeah, so I saw it online and I found it quite interesting to have a 24-hour gym, because most of the time I spend it in school, so during midnight I will be going to gym…So I actually went to sign up. It’s quite costly actually, but if I were to look for a new gym, usually the flat rate is 88 per month…So I saw this discount where they have a new gym opening. So, I just went to sign up. It’s 68 per month, yeah, quite cheap. – G02, male, 19, Information Technology
T2·2 Exposure to government health campaigns on social media	I used the HPB [Health Promotion Board] step tracker thing but only because it gives you NTUC vouchers [participants laughing in the background] and then I would just fake the steps by shaking it. – B03, male, 22, Law
	Yes. It’s mainly for the rewards, like [another participant] said as well. So, because I was thinking like, definitely, you’re bound to walk every day, no matter how little you walk. So eventually you do… Can clock some steps. So, like you mentioned, there’s a total of seven tiers as well. I think I only managed to get about four or five because you need to consistently clock on average 10 000 per day. But, yes, since I’ve grown accustomed to it, I just keep on wearing it lah. Yeah. But the numbers of the steps themselves don’t really matter to me. – B05, male, 21, Accountancy
	The healthy SG, yeah. Healthy365. That’s a lousy name for an app. [participants laughed] But I think it works because of the monetary incentive. I found myself jogging more because I wanted to hit the target… I saw my parents using, like walking more because of that as well. They are very *nua* [Singaporean slang for “lazing around”] people. – D04, male, 24, Mechanical Engineering
	For me it’s like uh, okay, you know those like sponsored ads on Instagram, or something. So, like sometimes ActiveSG [a Singaporean Sport portal with sports news, events calendar as well as facilities and coaches directory for everyone to watch and play sports; available as a mobile application] they will come out, something like uhm, spend your credits today or something. So, for me it’s like a reminder lah, like, I need to spend my $100 free credit ah… I think they do like sponsor ads, yeah. ’Cause I’m very sure I didn’t follow them [on social media] – D02, male, 22, Mechanical Engineering
	I do use [ActiveSG] to book badminton courts, because I’ve been doing badminton since young. So actually that’s, that’s what I find it really useful, and it’s very cheap to grab one court maybe like $5 for one hour, so they give you the $100 free credit so you can book like quite a number of courts. Yeah, and then, um, tsk, uh I think, uh I think having like monetary incentive really does, uh, make people want to work out. But after that, for it to be sustainable right, people have to realize that uh, tsk, exercising must become a part of your lifestyle. Like you do it, not because uh, you want to earn money but because you know that it is healthy for you. Yeah, so hopefully by having all these monetary incentives you can actually motivate people to think in this manner. So I think that’s how it will eventually work. – D03, male, 24, Pharmacy
	[When] I’m looking for healthier choices. I actually use this quite a lot, the Healthier Choice sign. I think the government did a good job in implementing that. I actually look for that if I’m shopping for groceries, quite often, yeah. – D01, male, 21, Chemical Engineering
	Yeah. But, but then again, it’s like, it’s kind of scary too. I, I remember one that says that if you eat a slice of *roti prata* [South-Indian flat bread served with curry], you have to run, like, I don’t know. – D07, male, 24, Political Science Those are, those aren’t really helpful at all. [participants laughed] I mean, like, this pineapple tart, you can run for three days, it won’t go away. [participants laughed] And the thing is, they, they completely ignore, like, the calories you lose while you’re sleeping, that kind of thing… And they like to…. – D01, male, 21, Chemical Engineering Exaggerate ah. – D04, male, 24, Mechanical Engineering Fear monger. – D07, male, 24, Political Science
	I think for my family, like slowly we are becoming more and more, in a sense, health conscious. I don’t know, like, every time we go grocery shopping then we will always check the labels and things like that. I mean, it feel a bit like, over – it feels a bit too over-exaggerated sometimes, but I feel like it’s quite necessary, because even things like, yeah, granola bars and cereal, like, as long as there’s, um, like high fructose corn syrup, then that in itself is really not good, even though they like to package it is as, oh, something healthy, even if it’s like oat-based. My family also starts to, like, shift to, like, more healthier food choices, so for example like – ’cause like, my sister and I, we don’t really care, what my mum buys at the supermarket. We just eat. So, like, she’ll she change from, like, white rice to brown rice, but like we don’t – we don’t really mind. And also ’cause brown rice, it doesn’t taste as bad as it used to last time. My mum always says that she always – like, “why do you hate brown rice? It tastes exactly like white rice”, like, like she always – she always, like, tells my sister like that. And like bread in our house is no longer white bread. But, like, like, whole meal bread or, yeah, we try and, we try and… like, so, like, we just changed, like, what we already eat to, like, slightly healthier options, but we won’t, like, completely remove, like we won’t remove bread cause oh bread is like carbs or whatever. But we will still eat bread, but we change to, like, whole meal bread, yeah. – C03, female, 21, Business Administration
T2·3 Social media and peer influence	I don’t follow any food related accounts on Facebook or Instagram, but I see them on my feed, because people share them. And for Instagram I see it as well, because it appears on my explore page. – C05, female, 20, Communications and New Media
	Like the… I think like for social media they actually uh, they actually show you things that you search for? So, they’re kind of like tracking your preferences, so the more you search for food the more food they’ll show you. So it doesn’t really help if you’re, like, you’re not exercising and stuff, and they just keep showing you food and things like that. And like the workouts on Facebook, like I mean those that I’ve seen, are very simplified? They make it seem like you can work out anywhere, anywhere, those, those kind of things. But I, I tried. But it’s, it’s damn lame, like, it doesn’t even work… I mean, it’s just like push-up challenge thing. I think it’s a 30-day thing, and like every day you do one [more than the day before]. It doesn’t train me. It doesn’t do anything to me. – D07, male, 24, Political Science

#### Subtheme T2·1 Exposure to companies on social media

Participants were aware of how food companies used advertisements to influence their food and activity choices. They discussed social media influencers posting sponsored content, with some recalling experiences of friends who write sponsored posts, resulting in inadvertent exposure to food advertising due to their social networks. Perceptions towards sponsored posts tend to be negative as participants believe that the people who write sponsored posts are ‘*doing it for the money*’ (A04, female, 20, life science) or exposure. Moreover, most of the participants agree with the sentiment expressed by C08 (female, 23, Faculty of Arts and Social Sciences; undecided academic discipline) on commercial advertising campaigns, ‘*I think in general, like, commercials, all that, like, I think they’re just trying to lure customers. Yeah, it’s just a money-making business lah*.’. (Note: ‘*lah*’ is a commonly used particle, typically used at the end of sentences or words as part of colloquial Singaporean English (Singlish) whose meaning is different (can place emphasis, affirm, and dismiss what is said) depending on the tone, syntax, and context.) Thus, many participants actively browse past advertisements on social media unless they spot promotional deals such as discount codes and coupons.

Meanwhile, some participants install Adblockers on browsers to avoid seeing any advertisements. However, this does not mean that participants are entirely unexposed to commercial advertisements, as they will still be exposed to their friends’ posts and social media activity, which may also initiate a discussion or activity related to the advertised products and services. Nonetheless, social media continued to be a resource for finding promotional deals for products and experiences for many participants. However, personal interest, friends’ influence and promotional deals had more influence on whether they chose to engage with marketing campaigns.

#### Subtheme T2·2 Exposure to government health campaigns on social media

Government health campaigns on social media were met with mixed reception. One of the most discussed campaigns was the annual nationwide National Steps Challenge™ organised by the Singapore Health Promotion Board. Participants received free pedometers to track step counts and used a mobile application called ‘Healthy 365’ to record their step counts in exchange for incentives. Participants recalled that the campaign was heavily advertised on social media and in real life. Much of the discussion centred around barriers to participation, including technical issues with pedometers and the mobile application, and ways to inflate the step count to get the incentives. Whilst many participants stopped using the pedometer and mobile application after the incentives were discontinued, some participants continued their use as they got used to being more active. Overall, at least during the challenge, many participants and their friends and family took part in more activity.

Another government health campaign that was frequently discussed was the ActiveSG campaign. With ActiveSG, patrons can visit affiliated gyms at SGD2·50 (USD 1·88) per session and book recreational facilities at very low prices. Additionally, an annual monetary credit of SGD100 was provided by the government to use at these facilities^(30)^. Many participants recalled being first exposed to the campaign on social media through sponsored posts, which also served as reminders to use their credit. While some participants made use of the credit and facilities, others did not due to lack of time, poor accessibility, the gyms being overcrowded or having old equipment and the perception that social gatherings generally do not involve physical activity. Similar to the National Steps Challenge™, long-term maintenance of physical activity habits once gifted credit has been used was questioned.

Healthy eating social media campaigns hosted by the government also elicited critical responses. A campaign which required students to add a hashtag on social media in exchange for a SGD2 discount on a ‘healthier’ choice lunch item was faulted for not incentivising a wider range of more healthful meals or for discount redemption being troublesome. Participants who engaged with this campaign primarily did so for the discount, while others did not want to be affiliated with a health campaign on social media. Another heavily promoted health campaign on social media and at food outlets was ‘Healthy Plate’ where more healthful food portions were promoted. ‘Healthy Plate’ was also perceived as having limited success by participants as they tend to eat whatever portions they wanted and felt that at least on campus, they did not have many healthful options. General perceptions towards health campaigns amongst most participants concur with sentiments expressed by D06 (male, 24, sociology), ‘*I think sometimes like all these health tips, right, it’s a bit too, uh, like it assumes like you have a choice lah. But sometimes we are really just limited by our circumstances*’.

Participants largely appeared to trust information from government entities, such as the Singapore Health Promotion Board and Singapore Ministry of Health, referring to contents of health campaigns, or product endorsements as a benchmark for what they considered healthful in their discussions. However, at times, due to the way some health messages were framed and interpreted by the participants, some participants expressed apprehension about its veracity. For instance, participants voiced that caloric content of food items were inflated in a government-endorsed food-tracking mobile application to scare them into being more health conscious and were generally not helpful to them. Conversely, some participants talked about improvements in family members’ food literacy and preferences due to health promotion efforts. For instance, after one participant’s mother learned about healthful alternatives through government campaigns, the family’s diet incorporated these switches for more healthful meals at home, in addition to reading nutritional content labels. Consequently, that participant continues to follow these beneficial changes outside of the home as well.

#### Subtheme T2·3 Social media and peer influence

The influence of peers was evident by the narratives participants recounted on the types of content they were exposed to, engaged with and shared amongst their networks. While in some instances, posts by friends initiated or reinforced health-promoting behaviours, a more commonly shared experience was being tempted by shared posts with new and promotional food deals as experienced by F06 (female, 18, aviation management), ‘*Like yeah, you get tempted by your friends like Instagram post like, they’ll post like, uh, “try this place”, “it’s a new place”, then they post the food, then like, you [are] also like tempted to go and buy*’. Social media also provides an avenue to explore other content of interest as algorithms suggest content to users based on personal interests and their friends’ use. However, some participants shared that this may work against their goals of making healthier lifestyle choices. Nonetheless, responses of the participants suggest that they purposefully use social media to seek information on trends, experiences, promotional deals and recommendations within and beyond their immediate social network. Generally, shared promotional deals and recommendations often result in related social activities in real life, such as a group outing to visit a newly opened café offering promotions, demonstrating how shared information on social media influences food and activity choices in reality.

### Theme 3: Real-world family and peer influences on health-promoting habits

Apart from interactions on social media, participants discussed how real-life interactions with their social networks influence their perceptions and practices around food and physical activity. Friends and family can influence behaviour in several ways such as by providing information, serving as role models, shaping perceptions about body image and acting as arbiters of accountability. Illustrative quotes for this theme are presented in Table 4.

**Table 4:** Quotes for theme 3: real-world family and peer influences on health-promoting habits

Sub-Theme	Illustrative quotes
T3·1 Role modelling	At certain times, like, all your friends are like eating healthy, then, like, you are subconsciously, like, oh, okay, maybe I should, like, hop on the bandwagon and try it too, that kind of thing. [participant smiled] or like um, yeah, I don’t know lah. It’s just phases lor, yeah. – C05, female, 20, Communications and New Media
	Then for me with regards to exercise, occasionally I have those friends that – they will post their, like, workout videos, and like, oh cool, I wish I could be like you, but too bad I don’t have the self-control, time, or energy to do that. Yeah, although, like, uh I do kind of – like, as in I try to exercise, but I just – just fail lah. – C06, female, 20, Psychology
T3·2 Health literacy and practices	Because my friend, like, she is quite, like, a health conscious person. Then, like, the time, like, I had stayed over with her, then she show me her six packs [referring to her friend’s abs] …Then she say, like she – she achieved that ’cause she didn’t drink any sugary drinks lah. So I’m, like, very motivated, I’m like, okay. [participants laughed] – C08, female, 23, Faculty of Arts and Social Sciences; Undecided academic discipline
T3·3 Recognition, accountability, and body image	I play soccer sometimes also. Then uh, like for example, if my teammates notice that my stamina improved. Then they tell me like, “eh your stamina become better *sia*”, and say like, “good job” or something, then I’ll feel better lah. Then I guess that’s one of the benefits, rec - recognize your effort. Then like what he [another participant] said just now, sometimes my girlfriend will say, like, “wow, so fit”. Then whoa *shiok* [Singaporean expression for expressing admiration or approval] [other participants laughed]. – D06, male, 24, Sociology
	Like you see all these like clothes [on social media] and then you’re like, oh my gosh, if I could like be like that woman wearing that clothes. You see like these models and things like that. And then it’s like, um… Or I used to like want to like really like wear bikinis and everything, and stuff… But then like, um, sometimes I’m just like, yeah, I just feel fat so then I was like I will just exercise. But, um, this is very weird, but like honestly I think that like, um, my… like look-wise I’ve been less and less motivated to like exercise for looks because, um, increasingly I’ve been surrounded by people, like close… people who are close to me who accept me for who I am. [participants laughed] So like they are very like accepting and like they’re very loving, and like they don’t make me feel inadequate. So, I don’t feel inadequate, so I don’t work, so I don’t like engage in physical exercise to improve my looks ’cause I don’t think that my looks are that bad. – A08, female, 20, Political Science
	I think there was – there was this period of time like in school when, like, I think it was very stressful and stuff, so my friends and I, like, we went to the gym every day, and then, like, we make sure, like, we kept each other accountable about what we ate… Like, let’s say in the canteen, like, if we are, I don’t know, if we are – let’s say we go to the [Mixed Vegetable Rice] stall then, like, we see what [is available], and we’ll [be] like, “yeah, no fried food okay?”, that kind of things… And then, like, uh yeah, like, you ask for less rice, or, like, brown rice, that kind of options, yeah. So, we were just – like, I don’t know, just keep in check on one another. – C03, female, 21, Business Administration
	When I go to my grandma’s house, she never fails to tell me that I get fatter. Like, every two weeks, like every two weeks when I visit her, and then like, my aunts or my grandma or my mum, like, they will just like comment. ’Cause like, now I don’t – like now that I don’t live at home, like, I think it’s more obvious, like, to my mum also… She will notice that there are, like, stark differences… I’m usually in hall every day. So, people in hall wouldn’t be able to tell me, like, anything, because they see me every day, but like, when I go back home, everyone’s like, “Hey”, I’m not sure why or how come [I’ve gained weight], so I think that’s like a very big problem for me, yeah – C04, female, 19, Psychology
	I’m a guy… My, my parents always tell me, like, “eh, you’re a guy, you must eat heartily, must eat a lot”. You know, eat a lot of rice, then you will grow taller, you will grow bigger. Yeah, but then I know for a girl it would be different lah, like my sister, she, it’s different for her. Like they tell her, “Hey, why do you eat so much? Uh, why you eat so much white rice?” – D06, male, 24, Sociology
	I think it’s more the people. Um, because, like, for instance, okay, like myself, if I go for a run, right, I find it very difficult to run longer distances by myself, compared to when I’m running with a friend. And, like, the rate of my – the speed at which I run is, like, faster when I run with a friend. I don’t know why. It’s, like, I just feel less tired. And like, we’re not even talking, but it’s, like, I just feel, like, I cover more distance in a shorter amount of time. But if you do it yourself, then it’s, like, it just feels like it takes forever, and, I don’t know, maybe it’s because there’s that sense of like, competition, so you’re, like, oh, I – like, oh don’t want to lose face, like oh, I don’t want to like yeah, trail behind and stuff lah. – C05, female, 20, Communications and New Media
	I hate going to the gym. I don’t like it. I don’t know. I just feel like all the gym rats are there. [other participants laughed] It’s very intimidating [another participant says ‘yes’ in the background, agreeing with A08’s statement] and like I… Hmm. Some of the machines are so weird [other participants laughed] and I’m not sure what to do with them. I’m not sure if I’m doing it right. Then I’m like… I’m, I’m not sure if like my… what’s like… I don’t know. What do they call it? Like whether their posture is correct. Oh, whether their form is on point or not [another participant says ‘oh yeah’ in the background, agreeing with A08’s statement], right. Then like there, there will be people who are more experienced who are judging me and my bad form. And like, I don’t know. There’s too much like social pressure and like it navigates the whole situation. Like it feels too social for me. Like I prefer exercising alone personally. So, like, um, sometimes like asking a friend is like the person will probably have to be a very good friend to watch me die. Because [other participants laughed] I will just die. Like, yeah. And then like I prefer being alone and then like, like that moment when like your… the exercise takes over and then like you can’t think of anything. You can only focus on breathing because you’re dying. [other participants laughed] Like I think that’s quite nice. [other participants laughed] Yeah, like I think is… Yeah, I don’t know. – A08, female, 20, Political Science

#### Subtheme T3·1 Role modelling

Peers who incorporated healthy meals and exercise into their daily routine were often represented positively by participants as being a ‘role model’ or ‘disciplined’. When friendship groups are interested in health-promoting practices, participants may also find themselves eating healthier. Likewise, when friends are also interested in exercise, they can influence participants as D05 (male, 21, economics) comments, *‘I think friends are important uh. Like if your friends exercise then you nothing to do, “Okay lah, I’ll follow you ah”.’* While this was true for several participants, there are also those who remain unmotivated due to personal barriers.

Peers were variably described as providing motivation to engage in healthy behaviours or serving as sources of temptation. While peer behaviours were sometimes described as being aspirational, incongruity between personal aspirations, perspectives and practices was sometimes observed. For instance, while D07 (male, 24, political science) thinks that having free time means that you can have a healthier lifestyle and that it is a sign of discipline to maintain these practices he also thinks that *‘eating healthy is a bit, is a bit sad? Like everyday life is so stressful and you’re eating healthy, it’s kind of not helping?’*.

In other situations, peers may entice individuals to make unhealthy food choices by saying words to the effect of ‘It’s just once in a while’. Some participants reported strategies to maintain healthier food practices even when friends had different food preferences such as eating before the gathering or choosing a healthier alternative when dining with friends at fast-food restaurants. However, most of the time, participants found it hard to resist the temptations of enticing food and their friends’ invitation.

#### Subtheme T3·2 Health literacy and practices

Participants described how family members’ health literacy and practices can influence their behaviour. For example, some participants explained that they have no choice but to eat late at the behest of their family members, a practice which they feel adversely affects health. Similarly, peers may also be a source of information on health-promoting practices when they are interested in health-related information. For instance, a participant became motivated to curb her sugary drink intake when she found out that her physically fit friend did not drink sugary beverages to maintain her figure. Though at times, some participants may oppose their peers’ ideas on health, such as with G01 (male, 19, information technology) who is interested in bodybuilding, ‘*I have talked to, like, a few of my female friends who are, like, into dieting and stuff also. So, like, um, what usually their mind-set was that lesser calories equals to a healthier diet so that they don’t gain weight and stuff, yeah. So, like, this doesn’t really make any sense lah because… they just have like this very broad idea that calories is bad, so…* [trails off]’

#### Subtheme T3·3 Recognition, accountability and body image

Positive self-image and personal development were partly attributed to having a supportive group of friends who motivated, validated and recognised young adults’ health-promoting efforts. In general, having supportive friendships improved participants’ body image. Participants who lost weight or kept up healthy practices attributed it to a supportive group of friends that is respectful of participants’ individual preferences. Participants also felt positively about these friendships especially if they have had past struggles with exercise and weight gain. In general, joining sports clubs, interest groups, having a goal or joining competitions also encouraged more exercise. Male participants reported joining friends to train for annual fitness tests post-conscription. This demonstrates how friendship groups can have considerable influence, holding members of the group accountable for their health behaviours.

Outside of friendship groups, some participants described their family and relatives as ‘*mirrors*’ that compel them to reflect on their appearance. Such comments may have an impact on how participants perceived themselves, especially if the comments are negative. There was a gendered aspect on food practices in the family, in particular with regards to portion sizes, with young men being encouraged to eat more and young women cautioned against eating large servings. Gendered perceptions were also visible with regards to exercise as women remarked that they preferred to exercise with friends with whom they felt comfortable revealing their untidy selves, especially if they struggled with exercise. Most participants, both men and women expressed that they would be more likely to exercise, and to do so more vigorously, when accompanied by a friend.

### Theme 4: Role of norms

The activities that participants took part in and accounts of their peers’ behaviours revealed social norms that influence daily living and health-promoting practices. Illustrative quotes for this theme are presented in Table 5.

**Table 5:** Quotes for theme 4: role of norms

Sub-Theme	Illustrative quotes
T4·1 Student priorities – studying and socialising	And what makes it [physical activity] difficult, there are so many things that make it difficult. Like what [another participant] said lah, like she’d rather watch a drama. Like, why go to the gym and, like, tire yourself out more when you can, like, relax and watch a drama and stuff. Yeah. – C05, female, 20, Communications and New Media
	I’m not getting enough sleep, I guess, so to fit in my gym into my schedule, usually if I were to go home around 10 plus? Yeah, I will be gyming around 11 onwards, so usually I would get around four hours of sleep to five hours of sleep. – G02, male, 19, Information Technology
	I mean, you only get three to four years in Uni, why not just play and enjoy it first before you actually go out and start working then you won’t have these kind of supper nights anymore… while you are still in Uni, yeah, just go along with it lah. – B02, male, 22, Economics
T4·2 Gym culture	Like those uh, like, I know the few guys on Instagram, so they’re just all the time like admiring. Like, like they started using words they’ve never used before, and it feels very… weird. – D01, male, 21, Chemical Engineering Yeah. I just can’t stand guys who do that. – D07, male, 24, Political Science Goes off into a world of their own. [Participant laughed] … Then it sort of estranges [everyone] away. – D01, male, 21, Chemical Engineering
	There is one more reason why I don’t go uh gym because that time after a long time never go to gym I went with my uh a bit oversized friend, ’cause she was determined to lose weight. Then we went to the gym right, there was like these guys who were like laughing at her [other participants saying “so bad” together] and we felt so offended for her. So apparently after that like she didn’t want to go to gym anymore. Then also, we also like feel bad already and we don’t go gym already. – E02, female, 19, IT Service Management
	I have friends who like don’t like to gym, because they feel very conscious of the people around them. And then for me that was how, like, I felt when I first started going to the gym, and like – there’s a – like, last time it used to be, oh, only, like, guys go to the gym, like, girls don’t really do it that kind of feeling. Girls just, I don’t know, jog on the treadmill, but you don’t touch the weights, yeah. But then, um, subsequently I realised, actually that’s not really the case ah. Like in fact, like guys will be more conscious of girls being in the gym than – because they want to look good. But also, at the same time, like, people actually don’t really care what you’re doing, about what you do ah, people aren’t looking at, like, you, they are looking at, like, they prefer to look good themselves. [fellow participants laughed] Yeah they want to, yeah they rather make sure that they are, they are the ones looking good, as opposed to, oh, how you are doing. Yeah, that kind of feeling At least that’s what – like, that’s what I personally feel uh, yeah. – C03, female, 21, Business Administration

#### Subtheme T4·1 Student priorities – studying and socialising

Valuing academic achievements as a priority appeared to be a common mindset in most men and women, with a male participant, who served in the nationwide compulsory conscription, quoting ‘*Now I am a full-time student, not a soldier*’ to demonstrate his priority as a tertiary student taking precedence over other activities. Along with studies, other priorities may include part-time jobs that the students take on to support themselves. Several participants described prioritising time to rest or study over taking part in exercise. Some participants who chose to take part in physical activity noted that it cuts into their time for sleep.

Socialising was also perceived as a priority and participants reported spending time to form and maintain connections and friendships during this phase of life. Thus, peer influences on dietary behaviours may be particularly pronounced as commensality or shared eating facilitates occasions for young adults to forge new and strengthen existing friendships. For instance, NUS participants who lived on the university campus expressed that it is a norm to have late-night suppers with other students, especially as first year students. These suppers provided a way to socialise and form friendship groups despite the unhealthy practice of eating calorie-dense foods, which were perceived as being the only options available at late hours. Upon forming friendship groups, participants reported feeling less pressure to join suppers, which were positively viewed by some participants as saving time and money.

Thus, these priorities influence the amount of time participants have to take part in health-promoting activities, especially when they often prioritise studying over exercise and eating healthfully. This can result in phases of inactivity and unhealthy eating during exam periods and more activity and healthier eating during non-exam periods.

#### Subtheme T4·2 Gym culture

Some virtual norms and behaviours for exercise are also enacted in real life. Many participants described online and real-life gym culture as being showy, obnoxious and alienating to those who do not take part. These negative social experiences reinforce barriers to physical activity. Many women described gyms as male-dominated spaces, resulting in a perceived hostile environment for women, especially if they were to exercise alone. Participants also shared negative experiences such as when their friend was fat-shamed by other patrons, which resulted in participants never going back to the gym. However, some women shared experiences where they felt better able to exercise in the gym if they went with a friend or once they noticed that fellow gym-goers were more focused on their personal performances.

## Discussion

This study explored young adults’ perspectives of social environments, both real and virtual in relation to their food and physical activity choices. Our results suggest that social media influences dietary and activity choices through pathways of meeting physiological and social needs, creating or reinforcing norms and serving as sources of influences from peers, family and commercial and governmental entities. Real-world social connections similarly served as sources of information, role modelling and determinants of body image. Participants seemed to navigate these 2 worlds in a complementary manner and when presented with conflicting views such as whether a popular café has tasty food, the opinions from real-world sources, i.e. close social networks, generally took precedence.

Comparable to other studies^(10,31)^, the high prevalence of our participants’ social media use was driven by the need to connect with others, express themselves and seek validation. Social media complements traditional ways of bonding with friends and families and facilitates new connections with others on a virtual platform that fuels interactions with online content. Social media is also an efficient platform to share and promote information, create social capital and set the scene for social norms that persist in real life, such as gym culture. Simultaneously, social media was viewed as a platform that informs users of current trends, a way of seeding trends and creating normative behaviours. In general, the types of information viewed and shared reflect the interest of individuals and their social networks, especially as social networks tend to be homophilic since similarity breeds connection^(32)^. However, the high frequency of use also suggests that there is an underlying ‘fear of missing out’ on trends and events^(33)^.

Consistent with results from a study in European adults, our participants also reported using sources apart from social media as alternate channels of information^(34)^. While our participants valued content from reliable sources, they also admitted to instances where they shared content based on the headline without perusing the content. Experimental evidence suggests that apart from the perceived credibility of the news publisher, social network tie-strengths can also influence the credibility of the news shared, with closer ties being more influential^(35)^. In line with these findings, we observed that our participants were more likely to trust shared food and product recommendations from their close social networks at face value rather than those from social media influencers. Nevertheless, the source of information was still important with governmental sources being regarded as more legitimate and less likely to be fact-checked by participants as compared with unreferenced articles of interest.

Commercial marketing campaigns often target young adults with aims to promote sales, recognition and brand loyalty – often successfully^(36)^. Our results suggest that marketing campaigns that use price promotions, leverage on the latest trends and present trusted sources for recommendations were more successful amongst young adults. These campaigns seemed to initiate or influence more food-based rather than activity-based social occasions. Virtual recommendations of eateries or foods triggered activities in real life and were followed up by sharing these activities on virtual spaces, thus fuelling trends. Several contextual factors may act together to facilitate this in Singapore, as elsewhere. The wide range of eating outlets with a competitive retail food industry^(37)^ means that various foods are available to suit a range of budgets and there are usually new places to discover. With over 75 % of adults eating out daily, eating out is socially acceptable and can be viewed as normative behaviour in Singapore^(3)^.

Clever marketing by food companies by use of social influencers and native advertising also makes it harder to distinguish paid advertisements from genuine recommendations^(38)^. Our participants were aware of the persuasive elements of social media algorithms and marketing content. However, social media engagement seems to sustain trends that are stimulated in part by the ‘fear of missing out’. Simultaneously it provides content to share with social networks and opportunities to participate in new experiences with their friends in real life, thus facilitating the spread of advertised content. Successful social marketing of modern manufactured foods and meals is closely related, historically at least, to the epidemic of obesity. Consequently, this has included a muddying of the water for consumers by appropriation of the language used for health promotion as the term ‘healthy’ has no real meaning when used in food marketing and where pursuit of health through healthful eating is often confounded with, or limited, to slimming^(39,40)^.

Unlike industry marketing campaigns, which were generally viewed with a level of mistrust, participants largely trusted government-endorsed media, a finding that is consistent with survey data^(41)^ and bodes well for receptivity to health promotion efforts. Although there were no clear answers on how to improve young adults’ engagement with government health campaigns on social media, participants were receptive to government health campaigns in terms of viewing promoted content before deciding on whether to take part in promoted activities. While concerns about programme sustainability were raised, physical activity-related health campaigns seemed to have successfully used incentives to facilitate engagement to some extent. Our results diverges from a study which found that Scottish young adults viewed health campaigns as unengaging due to dissemination methods and messages that neither help overcome perceived barriers to health promotion nor were tailored to young adults^(42)^. Though, as suggested from our findings, improving some aspects such as the naming and usability of mobile applications, ease of access and engagement to promoted activities and the framing of health messages may help improve engagement for government health campaigns directed towards young adults.

Similar to young adults in the USA^(43)^, our participants were also exposed to a wide range of diet and exercise-related content on social media which rarely resulted in them taking part in these activities in real life. Posts related to food were commonly shared, suggesting that young adults are highly interested in food-related topics. However, shared posts were mainly related to eatery or food recommendations which tended to promote high-calorie foods, presenting contradictory messaging to healthy eating campaigns. These observations are consistent with those of Holmberg and coworkers who found that Scandinavian teens typically shared calorie-dense, nutrient-poor foods on social media^(44)^. In contrast to commercial food posts, and resulting real-life activities, our participants expressed reluctance to share physical activity posts of their own achievements. The reasons for this hesitancy were similar to those observed amongst young adults in the USA who did not wish to post physical activity-related information due to a perceived lack of interest from their peers, not wanting to appear annoying and perceiving physical activity as a personal activity^(45)^. Likewise, although posts related to physical activity promotion received more likes than posts on health education amongst Norwegian social media users, they were less likely to receive comments and shares^(46)^. Similar to a study from the USA, we found that while peers’ physical activity posts can sometimes be motivational, comparing their appearance or activity level with those of the post can lead to some participants feeling negatively about themselves^(47)^. Thus, due to their interest and use of social media, shared posts of young adults seem to compete with the dissemination and actualisation of health-promoting information and activities.

As young adults spend a lot of time with their social group, group preferences also have a marked influence on whether social media content affects lifestyle behaviours. This prioritisation of socialisation could be due to transitions in young adulthood where students shift from secondary schooling to an institute of higher education where in addition to students acclimatising to their new circumstances and studies, they also experience a need to form and maintain social bonds whilst they navigate this stage of life^(4,9,10)^. Similar to young adults in Belgium^(13,14)^, our participants prioritised studying and social occasions over other activities, including physical activity and healthy eating. The role of commensality as a mechanism for facilitating the start and maintenance of new and existing social bonds is well recognised^(19,48)^. Indeed, in our study, several discussions of social activities centred around eateries, many of which were promoted on social media platforms. Further, social eating is related to enjoying foods that are perceived as being tasty, which are usually foods that are low in nutritional content^(49)^. Taste and cost are important drivers of food choice in budget-conscious young adults^(14)^, and may have contributed to sharing of food posts that are related to these aspects.

The types of food-related posts may also be indicative of the eating behaviours of young adults^(50)^, especially as they can model their eating behaviours on those around them^(14,19)^. Consequently, young adults were more likely to eat calorie-dense foods if their friends did^(19,51)^ and these types of foods are also more likely to be promoted on social media^(44)^. Hence, the prioritisation of socialising can work against healthy eating. Although commercial advertising was viewed with cynicism, they were nevertheless persuasive and participants actively accessed social media for food-related promotional deals. Similarly, studies in China and Indonesia showed that the abundance of food-related posts on social media, such as price promotions, snacks and brand name food products contributed to increased intake of unhealthy foods amongst their youth^(52,53)^. Our findings emphasise the need for regulating the marketing of unhealthy foods on digital platforms. As young adults tend to view their close social networks as particularly trustworthy sources of information, measures to regulate the re-posting of unhealthy commercial food advertising to limit inadvertent exposure to unhealthy food marketing are important. In Singapore, whilst guidelines to limit unhealthy food advertising to children of 12 years or younger exist, these are limited in scope^(54)^. Regulatory measures related to mandatory front-of-pack labelling and advertising prohibition for unhealthy beverages are being implemented and extending similar measures to food would be of value to consider^(12,55)^. Additionally, supporting the use of healthier ingredients by the foods service sector^(12,55)^ can be further strengthened.

Similar to young women in the UK^(16)^, our participants often felt judged for their appearance and ability whilst participating in physical activity. Further, our participants were subjected to gender norms regarding dietary intake which can influence their self-image. Studies have found body image, food preferences and practices to be highly influenced by family^(56,57)^. The combination of these gendered perceptions presents limitations on the circumstances under which women feel comfortable taking part in physical activity. Further, like their counterparts in the USA^(43)^, our participants perceived gym culture presented on social media and in real life as obnoxious which deterred some women from going to the gym. Negative perceptions of gym culture may have also contributed to participants’ reluctance towards widely posting their own physical activity achievements as people who did so were perceived as being annoying or showy. Although changing the narrative around physical activity in the social media sphere may promote more physical activity as social media users who posted physical activity related posts were more committed to doing physical activity and had higher levels of physical activity^(45,58)^.

Overall, maintaining health-promoting activities was commonly perceived as challenging, unless the young adult has incorporated these practices into their daily life. Taking part in these activities may also form a part of a participant’s identity as a health-conscious individual. However, most of the participants who tried to incorporate exercise into their routines were likely to decrease time spent on studies, or more commonly, lose sleep. Nonetheless, our participants commonly perceived peers who manage to maintain health-promoting practices as role models which can help encourage the maintenance of these habits. These views were also expressed by middle-aged Canadian men, suggesting that these perceptions could be common amongst a broad range of age groups^(59)^.

Our study also demonstrated that virtual and real-life social environments can motivate individuals and their social networks to take part in health-promoting practices, although strong support is needed from their real-life social networks for these to be maintained. This is consistent with studies showing how supportive family and friends have a major influence on increasing physical activity and healthy eating^(13,51)^. However, some participants who have supportive and accepting friend groups may not feel the motivation to take part in health-promoting activities because their currently comfortable social state supports continuing their less healthful behaviours. Ultimately, the interactions that take place between virtual and real-life social environments can influence lifestyle habits that take place in real life and affect health-promoting practices. The social media environment has massive potential to generate global changes in attitudes, among and driven by young people. Social movement for climate change, or against US gun laws, are recent examples, and the opportunity clearly exists for young people to harness social media to combat obesity and diet-related chronic diseases^(8)^.

### Strengths and limitations

This study is one of the few studies on understanding digital media influences on Asian young adults and is the first to investigate the influence of the social environment on young adults’ food and activity choices through a qualitative approach in an Asian context.

The use of qualitative methods allows in-depth insights into the research topic and the exploration of the factors that influence young adults’ dietary and physical activity practices. The inclusion of young adults, both men and women, from various academic backgrounds provided diverse perspectives to be explored. Further, the use of focus groups allows for interactive discussions which more closely simulates real-life conversations as compared with one-on-one interviews, and can present a broad range of viewpoints^(22)^. Conversely, participants with contrary views may not be able to express their real thoughts in group settings. However, efforts were made by the facilitators to create inclusive environments. Study rigor was also improved by pilot testing the interview guide, coding by 2 authors (JL and ZT) and discussions about FGD content between JL, ZT, and SAR to maximise reflexivity. Due to the nature of qualitative methods, sample sizes were smaller than quantitative studies, however, as additional topics were not raised in the last few discussions, we deemed thematic saturation to have been achieved. With limited literature concerning the social influences on young adults’ food and activity choices in Asian countries, our focus was on exploring the range of opinions, ideas and themes which help elucidate and conceptualise factors of social influences^(60)^. Discussions about FGD content between JL, ZT and SAR also helped mitigate issues related to thematic saturation, such as reflexive discussions on collated data and derived themes. Our study does not consider the perspectives of young adults who are not studying at tertiary institutes. However, a majority of young adults in Singapore do attend a tertiary institution, with 80·4 % of 25–29-year-old Singapore residents attaining a university degree or a diploma and professional qualification in 2020^(29)^. Also, as most participants were Singaporean Chinese, reflecting the ethnic mix in Singapore, the findings may not all be generalisable to other populations.

The personal interests and backgrounds of the participants may have affected the content of the focus group. For example, participants from health-related disciplines could have been influenced by course content, resulting in leading discussions or overcontribution. This issue was mitigated by having focus groups with students from mixed disciplines and facilitators ensuring that each participant had time to express their views.

Self-reported information such as height, weight and food choices were not verified by objective measures. Our study sample had a lower proportion of participants with high-risk BMI of ≥ 27·5 (6·5 %) as compared with national statistics of 13·1 % in adults aged 18–29 years^(7)^. Although students were asked about their dietary choices, without tracking what participants actually ate, their nutritional intake was not measured. A previous study found that Singaporean university students were not eating healthfully^(2)^ and this was also implied by the types of social media influences and resultant food choices that the participants discussed in our study.

## Conclusion

Overall, our results highlight the growing influence that social media and digital social interactions have on young adults’ dietary and physical activity practices. The frequency of social media use is unlikely to decrease as it is an easily accessible medium and meets the multiple social, informational and utilitarian needs of young adults. The type of content accessed on social media also reflects the interests of individuals and their friendship groups, which influences their choice of real-life social activities and practices, creating and reinforcing trends in virtual spaces and real life. Tapping into the needs of young adults on social media, commercial food marketing campaigns utilising limited time offers, novel flavours or price promotions are often used to generate interest in trendy and affordable foods or eateries. This translates into food-based social activities in real life and competes with government health promotion messaging which is viewed as trustworthy but seems to be less persuasive. Prioritisation of studying and socialisation, and unhelpful cultural norms including those of gym culture in real life, also present important challenges to healthful behaviours. The presence of close and supportive social networks can also help overcome perceived barriers.

Taken together, our findings support the growing call for regulating digital marketing of unhealthy foods^(12,61)^ and emphasises the importance of the food service retail sector’s contribution to the dietary behaviours of young adults. Regulatory approaches, such as those addressing barriers towards access and affordability of healthy food items and limiting the availability and accessibility of unhealthy food, are also important to improve dietary behaviours in young adults^(12,51)^. Our findings also suggest that marketing strategies that meet young adults need for finding affordable food options, fitting-in and providing opportunities for socialising are particularly effective, and this can be informative for the design of government health promotion campaigns. Mitigating the negative perceptions of gym culture, addressing gendered norms around physical activity, and promoting the incorporation of physical activity in social gatherings, as done with food may be ways to encourage physical activity in young adults. Given the limited success in existing population-level obesity interventions, co-designing health campaigns with young adults for improved engagement and efforts to understand and improve digital platforms to support healthful choices^(8)^ presents a valuable area of future research.
